# Illuminating ALS Motor Neurons With Optogenetics in Zebrafish

**DOI:** 10.3389/fcell.2021.640414

**Published:** 2021-03-18

**Authors:** Kazuhide Asakawa, Hiroshi Handa, Koichi Kawakami

**Affiliations:** ^1^Department of Chemical Biology, Tokyo Medical University, Tokyo, Japan; ^2^Division of Molecular and Developmental Biology, National Institute of Genetics, Mishima, Japan; ^3^Department of Genetics, Graduate University for Advanced Studies (SOKENDAI), Mishima, Japan

**Keywords:** RNA metabolism, phase transition, neurodegenarative disease, optogenetics, protein aggregation

## Abstract

Amyotrophic lateral sclerosis (ALS) is a fatal neurological disorder characterized by progressive degeneration of motor neurons in the brain and spinal cord. Spinal motor neurons align along the spinal cord length within the vertebral column, and extend long axons to connect with skeletal muscles covering the body surface. Due to this anatomy, spinal motor neurons are among the most difficult cells to observe *in vivo*. Larval zebrafish have transparent bodies that allow non-invasive visualization of whole cells of single spinal motor neurons, from somas to the neuromuscular synapses. This unique feature, combined with its amenability to genome editing, pharmacology, and optogenetics, enables functional analyses of ALS-associated proteins in the spinal motor neurons *in vivo* with subcellular resolution. Here, we review the zebrafish skeletal neuromuscular system and the optical methods used to study it. We then introduce a recently developed optogenetic zebrafish ALS model that uses light illumination to control oligomerization, phase transition and aggregation of the ALS-associated DNA/RNA-binding protein called TDP-43. Finally, we will discuss how this disease-in-a-fish ALS model can help solve key questions about ALS pathogenesis and lead to new ALS therapeutics.

## Introduction

Amyotrophic lateral sclerosis (ALS) is a fatal neurological disorder in which motor neurons in the brain and spinal cord are selectively degenerated, leading to progressive muscle weakness. Approximately 1–2 individuals per 100,000 are diagnosed with ALS each year, with motor symptoms typically appearing in mid-adulthood (average age 55) (Taylor et al., 2016). About 10% of ALS cases are heritable (familial ALS) and linked to single-gene pathogenic mutations. The remaining 90% of ALS cases occur without family history (sporadic ALS) and are thought to involve multiple genetic mutations and/or environmental factors. While the root cause of sporadic ALS is unknown, its common pathological hallmark is deposition of ubiquitin-positive cytoplasmic inclusions containing aggregated forms of DNA/RNA-binding protein TDP-43, encoded by the *TARDBP* gene, in the degenerating motor neurons (Arai et al., 2006; Neumann et al., 2006; Mackenzie et al., 2007). Better understanding of the causes and consequences of TDP-43 aggregation will increase our understanding of ALS pathogenesis and aid in the development of therapeutics.

There is emerging evidence that an intricate intracellular network of biomolecular condensates or membraneless organelles underlies the physiological functions of cells, including neurons (Shin and Brangwynne, 2017). Many membraneless organelles are enriched in proteins containing regions of low sequence complexity, called low-complexity domains (LCDs) or intrinsically disordered regions (IDRs), which drive liquid–liquid phase separation through homotypic and heterotypic protein–protein or protein–RNA interactions (Mathieu et al., 2020). Aberrant phase separation of IDR proteins can generate solid-like fibers, which are candidate origins for irreversible aggregates that accumulate in neurodegenerative diseases (Lin et al., 2015; Molliex et al., 2015; Murakami et al., 2015; Patel et al., 2015; Zhang et al., 2015). TDP-43 is equipped with an IDR at its C-terminus, and has been identified as a component of membraneless organelles in the nucleus (Dammer et al., 2012; Gasset-Rosa et al., 2019) and cytoplasm (Dewey et al., 2011; Alami et al., 2014; Gopal et al., 2017; McGurk et al., 2018; Wang et al., 2020) under both normal and stressed conditions. The fact that ALS-associated mutations of *TARDBP* mostly occur in the IDR has led to the idea that pathological phase transitions of TDP-43 mediated by altered homotypic and heterotypic interactions through IDR contribute to ALS pathogenesis. Cytoplasmic aggregation of TDP-43 has been observed by overexpressing wild-type and mutant TDP-43 in cellular and animal models of ALS (Barmada et al., 2010; Li et al., 2010; Walker et al., 2015; De Giorgio et al., 2019; Watanabe et al., 2020). Since exogenously expressed TDP-43 can be toxic in the absence of TDP-43 aggregates, it is difficult to determine whether the TDP-43 toxicity stems from dosage increase, aggregation, or both (Barmada et al., 2010; Arnold et al., 2013; Asakawa et al., 2020). Therefore, dissecting oligomerization- or phase transition-dependent toxicity from overexpression-dependent toxicity has remained a challenging but important task in understanding the mechanism of TDP-43 neurotoxicity.

The zebrafish is a unique vertebrate model that offers access to spatiotemporal dynamics of disease-related proteins in *in vivo* motor neurons. With the high translucency of larval zebrafish body tissue, fluorescently tagged probes are visible in a single spinal motor neuron, allowing for direct visualization of specific proteins, organelles, and cytoskeletons in real time. In addition, genetic amenability and high genetic homology to humans have allowed for rapid establishment of stable zebrafish lines relevant for the functional exploration of ALS-related genes (Ramesh et al., 2010; Sakowski et al., 2012; Hewamadduma et al., 2013; Schmid et al., 2013; Da Costa et al., 2014; Armstrong et al., 2016; Lebedeva et al., 2017; Ohki et al., 2017; Lissouba et al., 2018; Shaw et al., 2018; Bercier et al., 2019; Bose et al., 2019; Kim et al., 2019; Asakawa et al., 2020; Bourefis et al., 2020). Recent studies have achieved optogenetic induction of ALS pathology by controlling the biophysics of disease-associated proteins with an unprecedented spatiotemporal precision by light illumination (Shin et al., 2017; Mann et al., 2019; Zhang et al., 2019; Asakawa et al., 2020). In this mini-review, we describe the skeletal neuromuscular system of larval zebrafish and optogenetic approaches for controlling *in vivo* phase transition of TDP-43 in motor neurons pertinent to the study of ALS pathogenesis.

### The Crystal-Clear Neuromuscular System of Zebrafish Larvae

**FIGURE 1:**
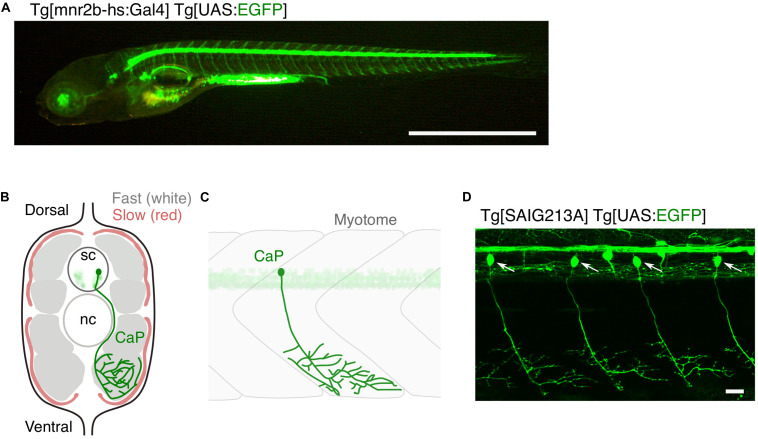
Large and small populations of spinal motor neurons can be manipulated with the Gal4/UAS system in zebrafish. **(A)** Tg[mnr2b-hs:Gal4]; Tg[UAS:EGFP] larva at 5 day post-fertilization. In the Gal4 driver Tg[mnr2b-hs:Gal4], the Gal4FF transcription factor (Asakawa et al., 2008) is expressed from the bacterial artificial chromosome (BAC) transgene carrying the *mnr2b* locus (encoding the Mnx-type homeobox protein, which promotes motor neuron differentiation) and drives expression of a gene downstream of upstream activation sequence (UAS) in the most of the spinal motor neurons. **(B)** Schematic illustration of a transverse section of the middle trunk of a 5 day-old wild-type zebrafish larva, with the dorsal side up. Fast-twitch muscle and slow muscle are shown in gray and red, respectively. A CaP innervating the ventral myotome is shown in green. sc, spinal cord. nc, notochord. Illustration modified from Bello-Rojas et al. (2019). **(C)** Schematic illustration of a wild-type CaP innervating the ventral myotome, from lateral view. **(D)** Among the spinal motor neurons, CaPs (arrows) are selectively labeled in Tg[SAIG213A] Tg[UAS:EGFP] fish. Bars are 1 mm **(A)** and 20 μm **(D)**.

Zebrafish grow to about 4 mm in length in the first 5 days of life, developing over 30 axial muscle segments in a fusiform, laterally compressed body (Schroter et al., 2008). In parallel, the spinal motor neurons are generated in the segmentally iterated spinal cord and innervate corresponding skeletal muscle segments (Figure 1A). A 5 day-old larva performs free swimming and is already an efficient visually guided predator (Budick and O’Malley, 2000), indicating a growing, but mature multimodal sensory-guided motor circuit. Generation of the spinal motor neurons largely ceases in the second day of development (Reimer et al., 2013), with 63–71 spinal motor neurons per spinal hemisegment present in the larval stage and remaining largely constant until the adult stage (van Raamsdonk et al., 1983; Asakawa et al., 2013; Svara et al., 2018). These motor neurons innervate peripheral muscles consisting of fast-twitch fibers that occupy majority of the muscle mass and slow-twitch fibers that cover the superficial muscle layer (Figures 1B,C). Spinal motor neurons are categorized into primary and secondary motor neurons, both of which start to differentiate at 1 day post-fertilization (Myers et al., 1986). There are five primary motor neurons in each spinal hemisegment (MiP, dRoP, vRoP, CaP, and VaP). These are the earlier differentiating population, possess relatively large-sized somas that align rostro-caudally, and innervate distinct areas of fast-twitch muscle fibers along the dorsoventral myotomal axis (Asakawa et al., 2013; Bello-Rojas et al., 2019). The remaining ∼60 neurons in a hemisegment are the secondary motor neurons that develop next: these possess relatively small-sized somas and innervate the deeper fast twitch muscle or superficial slow muscle fibers (Menelaou and McLean, 2012; Asakawa et al., 2013; Bello-Rojas et al., 2019). Overlapping patterns of innervation observed within and between these motor neuron types indicate polyneuronal innervation of muscle fibers (Lefebvre et al., 2007; Bello-Rojas et al., 2019). Zebrafish motor neurons, which vary in cell-size and physiology (McLean et al., 2007; Menelaou and McLean, 2012), might model selective vulnerability of motor neurons in ALS; large-sized, fast fatigable motor neurons are the most vulnerable to degeneration (Roselli and Caroni, 2014). Remarkably, unlike mammals, both larval (Ohnmacht et al., 2016) and adult (Reimer et al., 2008) zebrafish retain the ability to regenerate spinal motor neurons from the pMN progenitor domain in the ventral spinal cord after spinal cord injury or genetic ablation. This ability is worth further study for its potential contribution to regenerative therapy of motor neurons in humans.

A single motor neuron or a population of motor neurons can be non-invasively manipulated in zebrafish larvae with targeted expression of genetically encoded fluorophore and photo-responsive probes using the *cis*-elements of motor neuron-specific genes, such as Mnx-family homeobox genes (Wendik et al., 2004; Asakawa et al., 2012), and/or Gal4/UAS gene expression systems (Figures 1A,D; Zelenchuk and Bruses, 2011; Asakawa et al., 2013). Laser-assisted transection of a fluorescently labeled motor neuron (Rosenberg et al., 2012) was used to directly monitor, in real time, the sequence of events involving motor nerve degeneration following injury (Wallerian degeneration) (Waller, 1850), as well as the resultant dynamic interactions between the injured nerves and macrophages. Engulfment of the TDP-43-expressing motor neurons by microglia after UV-induced damage was directly observed (Svahn et al., 2018). Motor neurons can be more precisely manipulated by a method called optogenetics, in which genetically-encoded proteins change conformation in the presence of light (Deisseroth et al., 2006). Targeted expression and photostimulation of KillerRed (KR) generates reactive oxygen species (ROS) and induce motor neuron death, followed by microglial activation (Bulina et al., 2006; Formella et al., 2018). Motor neuron physiology can be manipulated by controlling membrane potential with photostimulation of the light-gated ion channels (Volgraf et al., 2006; Gorostiza et al., 2007; Wyart et al., 2009; Bernal Sierra et al., 2018; Antinucci et al., 2020). Further, development of motor neurons, such as axon guidance, can be optically controlled by light-activatable cytoskeletal regulator Rac1, promoting axon guidance of the caudal primary motor neuron (CaP) in the wild-type fish, as well as of plod3-/- mutant fish defective in axon guidance (Harris et al., 2020). In addition to these optogenetic methods, expression of appropriate fluorescent marker proteins enables the visualization of key subcellular structures of motor neurons, including pre- and post-synapses (Flanagan-Steet et al., 2005; Asakawa and Kawakami, 2018; Bello-Rojas et al., 2019), cytoskeletal components (Asakawa and Kawakami, 2010; Bercier et al., 2019), and mitochondria (Bergamin et al., 2016), in live animals.

### Optogenetic Induction of TDP-43 Aggregation in *in vivo* Motor Neurons

**FIGURE 2:**
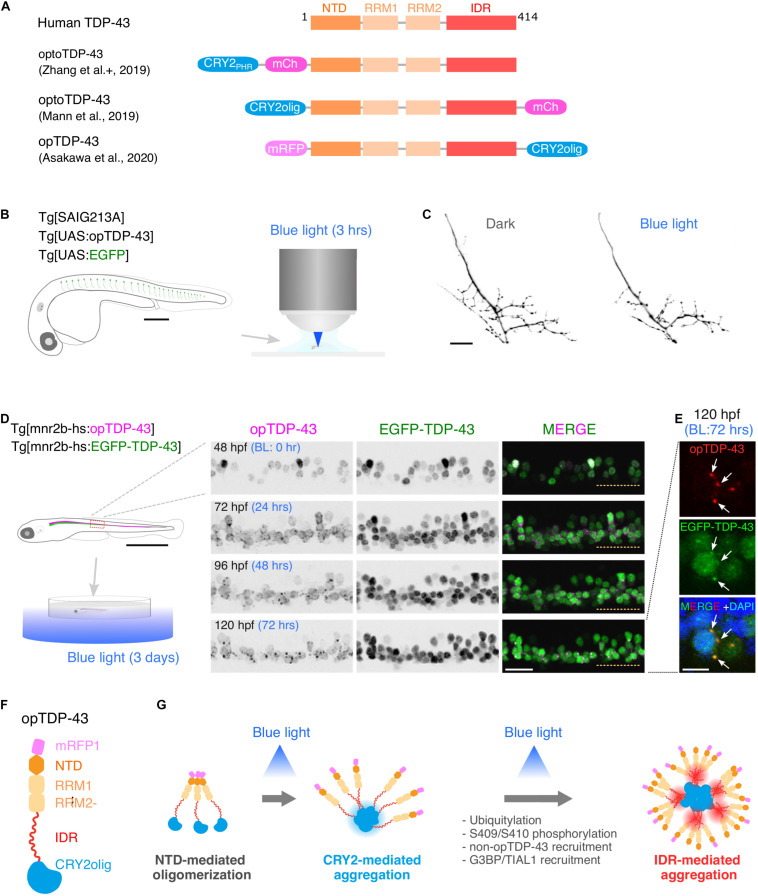
Optogenetic induction of TDP-43 aggregates in *in vivo* spinal motor neurons. **(A)** Structures of optogenetic TDP-43. The human TDP-43 (top) consists of 414 amino acid residues subdivided into the N-terminal domain (NTD), 2 RNA recognition motifs (RRM1 and RRM2), and a C-terminus intrinsically disordered region (IDR). The optoTDP-43 (Mann et al., 2019; Zhang et al., 2019) and opTDP-43 (Asakawa et al., 2020) constructs carry the CRY2-modules at their N- and C-terminus, respectively. mCherry (237 aa), mRFP1 (225 aa), CRY2_*P*__*HR*_ (498 aa), and CRY2olig (498 aa) not to scale with TDP-43. **(B)** An agarose-embedded zebrafish embryo expressing both EGFP and opTDP-43 in CaPs (Tg[SAIG213A] Tg[UAS:opTDP-43] Tg[UAS:EGFP] triple transgenic) is illuminated with a confocal blue laser light for 3 h, 28–31 h post-fertilization (hpf). **(C)** The total axonal length at 48 hpf was reduced in the CaP irradiated with the blue light (Blue light) compared to the CaP grown in the dark (Dark). The figure panels are adapted from Asakawa et al. (2020). **(D)** An unrestrained zebrafish larva expressing both opTDP-43 and non-optogenetic EGFP-TDP-43 was irradiated with blue LED light. The spinal motor column was scanned every 24 h for 3 days (from 48 to 120 hpf). The duration of the blue light illumination is indicated in blue letters. Horizontal dashed line demarcates the ventral limit of the spinal cord. The figure panels are adapted from the study by Asakawa et al. (2020). **(E)** Cytoplasmic opTDP-43 foci colocalize with non-optogenetic EGFP-TDP-43 (arrows) in the spinal motor neurons at 120 hpf in **(D)**. BL, Blue light. **(F)** Schematic drawing of opTDP-43 protein. **(G)** Blue light illumination drives CRY2olig-dependent opTDP-43 oligomerization and aggregation. A short-term illumination induces the oligomerization of opTDP-43, whereas a long-term illumination causes cytoplasmic aggregation of opTDP-43. Non-optogenetic TDP-43 is incorporated into the opTDP-43 aggregates [e.g., EGFP-TDP-43 in **(E)**] possibly through IDR-mediated intermolecular interactions. Cytoplasmic opTDP-43 aggregates are partially positive for immunoreactivities against ubiquitin, phosphorylation at S409/S410, and classical stress granule components (G3BP and TIAL1) (Asakawa et al., 2020). The bars indicate 250 μm **(B)**, 20 μm **(C)**, 1 mm (**D**, left), 20 μm (**D**, panels), and 5 μm **(E)**.

The optoDroplet approach, recently introduced by Shin et al. (2017), adopts the photolyase homology region (PHR) of Arabidopsis cryptochrome 2/CRY2 (CRY2_*P*__*HR*_) that self-associates upon blue light exposure (Bugaj et al., 2013) to induce oligomerization and phase transition of client proteins tagged with CRY2_*P*__*HR*_ (Shin et al., 2017). This technique has been successfully applied to TDP-43 in cultured cells (Mann et al., 2019; Zhang et al., 2019), and has been used to discriminate cellular events that are triggered by TDP-43 oligomerization from those triggered by dosage increase of TDP-43. In these studies, CRY2_*P*__*HR*_ or a point mutant version of Cry2 (Cry2olig) exhibiting enhanced clustering (Taslimi et al., 2014) was fused to the N-terminus (Mann et al., 2019; Zhang et al., 2019; Otte et al., 2020; Figure 2A). These optogenetic TDP-43 effectively displayed clustering upon blue light illumination, leading to cytoplasmic deposition of TDP-43 aggregates with the pathological signature of S409/S410 phosphorylation typically detected in degenerating motor neurons in ALS (Mann et al., 2019; Zhang et al., 2019), suggesting that the optogenetically induced TDP-43 aggregation mimics at least some of the TDP-43 pathology occurring ALS. Photostimulation of optoTDP-43 increases cell death rate in the cultured human neurons, with cytoplasmic shift and aggregation of optoTDP-43, demonstrating that optoTDP-43 phase transition is neurotoxic (Mann et al., 2019). The light-driven phase transition of optoTDP-43 does not recruit major stress granule (SG) markers, suggesting that TDP-43 is not a core component of SGs (Zhang et al., 2019). This result is consistent with the recent observations that TDP-43 that failed to be recruited to RNA-rich granules, such as SGs, is prone to aberrant phase transition (McGurk et al., 2018; Gasset-Rosa et al., 2019; Mann et al., 2019; Zhang et al., 2019).

A key step toward understanding of motor neuron degeneration in ALS is to induce TDP-43 oligomerization and phase transition in motor neurons and evaluate their cellular outcome in *in vivo* contexts (Asakawa et al., 2020; Otte et al., 2020). In zebrafish, expression of an optogenetic TDP-43 (opTDP-43), with which CRY2olig is harnessed at the C-terminus of TDP-43 (Figure 2A; Asakawa et al., 2020), can be targeted to CaPs among other motor neurons by using the prdm14-Gal4 driver (Figure 1C), enabling detailed analyses of opTDP-43 dynamics, as well as its cellular consequences, at subcellular resolution (Asakawa et al., 2020). opTDP-43 is primarily nuclear under normal conditions, as is the wild-type TDP-43, but dispersed throughout the CaP in response to blue light illumination for 3 h. Crucially, CaP axon outgrowth was halted even after the illumination ceased prior to cytoplasmic accumulation of opTDP-43 inclusion and the nuclear opTDP-43 localization was restored, showing that opTDP-43 toxicity precedes deposition of its cytoplasmic aggregates (Asakawa et al., 2020; Figures 2B,C). Live imaging analyses of the illuminated CaPs expressing opTDP-43 revealed an enhanced myofiber denervation frequency compared to the wild-type CaPs, underscoring the importance of normal TDP-43 phase behavior in formation and maintenance of neuromuscular synapses (Asakawa et al., 2020). The precise molecular mechanism of this oligomerization-triggered but aggregation-independent opTDP-43 toxicity is currently unclear. Another intriguing observation from the zebrafish model is that the light-dependent cytoplasmic opTDP-43 relocation occurred in the spinal motor neurons and the tactile sensing Rohon-Beard sensory cells in the spinal cord, but was almost absent in the embryonic epithelial cells and differentiated myofibers in a time frame examined (Asakawa et al., 2020). This observation suggests that the oligomerization-triggered cytoplasmic opTDP-43 relocation is a cell-type-dependent phenomenon (Vogler et al., 2018). Knowledge of the mechanisms underlying this neuron-specific cytoplasmic opTDP-43 relocation could aid in our understanding of cytoplasmic relocation of TDP-43 in the context of ALS motor neurons, whose mechanism is almost entirely unknown at present.

A major advantage of optogenetics is that it allows precise temporal control of a photo-responsive probe. In zebrafish larvae expressing opTDP-43 in most of the spinal motor neurons, the extension of the photostimulation duration from 3 h to 3 days resulted in the accumulation of cytoplasmic opTDP-43 aggregates in the motor neurons (Figures 2D,E; Asakawa et al., 2020). The cytoplasmic opTDP-43 aggregates contained non-optogenetic TDP-43 and were recognized to varying degrees by the antibodies against ubiquitin, phospho-S409/410, G3BP, and TIAL1 (Figures 2F,G; Asakawa et al., 2020), indicating that the opTDP-43 aggregates are heterogenous in protein composition and some species of the opTDP-43 aggregates recapitulate ALS pathology. While the pan-motor neuronal cytoplasmic opTDP-43 aggregation induced by the 3 day light illumination did not lead to overt motor deficit, the same set of conditions with opTDP-43 carrying ALS-associated IDR mutation (A315T) led to the failure to inflate the swim bladder and an impaired motor activity in a small population (13%) of larvae (Asakawa et al., 2020). Consistent with this observation, in fly, photostimulation of optoTDP-43 eventually leads to the formation of detergent-insoluble aggregates in the motor neurons that persist with age and cause larval and adult motor deficits (Otte et al., 2020). Overall, the temporally regulated induction of TDP-43 oligomerization and phase transition have demonstrated that TDP-43 exerts its toxicity through multiple mechanisms depending on its multimerization and phase status. Although a fraction of non-optogenetic TDP-43 was recruited to cytoplasmic opTDP-43 aggregates in the zebrafish optogenetic ALS model, most of it remained in the nucleus after 3 days of illumination during the larval stage (Asakawa et al., 2020). To determine if the cytoplasmic opTDP-43 aggregates eventually deplete the nuclear TDP-43 pool as observed in ALS cases, it is imperative to establish an illumination condition where the physiology of juvenile and adult fish is minimally affected by the blue light while the light-dependent opTDP-43 phase transition is fully controllable in time and space. In this regard, it is worth exploring other optogenetic probes for regulation of protein interactions that can be activated by different light wave lengths, such as a bacterial phytochrome-based probe sensitive to near-infrared light (Redchuk et al., 2020).

### Perspectives

The optoDroplet approach has explicitly demonstrated the causal relationship between TDP-43 phase transition and neurotoxicity *in vivo* in motor neurons. The accumulation of cytoplasmic TDP-43 aggregates is a hallmark of degenerating neurons in ALS. However, how TDP-43 is localized in the nucleus under normal conditions and the mechanisms by which it gradually loses its normal state and forms cytoplasmic aggregates are not clear. Thus, it is tempting to speculate that light-dependent opTDP-43 phase transition is a fast-forward replay of the changes occurring in TDP-43 in ALS, especially as TDP-43 dynamics is currently anatomically inaccessible in human motor neurons. Uncovering intermediate stages of the opTDP-43 phase transition would provide mechanistic insights into TDP-43 cytotoxicity and potential therapeutic targets. The illumination time-dependent formation of cytoplasmic opTDP-43 aggregates suggests that oligomerization and phase separation of opTDP-43 is tunable and can be used to reveal the temporal sequence of downstream events driven by pathological TDP-43 phase transition in the spinal motor neurons. This opTDP-43 phase transition can be targeted with subcellular resolution to explore spatial sources of TDP-43 toxicity, such as the nucleus, axon, and pre-/post-synaptic terminals. The ability to observe diseased motor neurons in a transparent animal also allows us to directly investigate systemic responses of neighboring cell types, including interneurons, myofibers, glial cells, and immune cells, to aberrant TDP-43 transitions occurring in the motor neurons. Moreover, since the IDR of TDP-43 is classified as a prion-like domain and TDP-43 has a prion-like aggregation-seeding ability (Nonaka et al., 2013), potential intercellular propagation of opTDP-43-driven TDP-43 aggregation could be directly tested within an intact CNS. Finally, we suggest that future studies take full advantage of zebrafish in a whole organism compound screening, advancing the opTDP-43-based zebrafish ALS model into a system for screening small molecules that mitigate toxic TDP-43 phase transition for developing effective ALS therapeutics.

## Author Contributions

KA wrote the manuscript and generated the figures, with inputs from HH and KK. All authors approved the submitted version.

## Conflict of Interest

The authors declare that the research was conducted in the absence of any commercial or financial relationships that could be construed as a potential conflict of interest.
